# A Systematic Review of Structural Health Monitoring Systems to Strengthen Post-Earthquake Assessment Procedures

**DOI:** 10.3390/s22239206

**Published:** 2022-11-26

**Authors:** Brian López-Castro, Ana Gabriela Haro-Baez, Diego Arcos-Aviles, Marco Barreno-Riera, Bryan Landázuri-Avilés

**Affiliations:** 1 Departamento de Eléctrica, Electrónica y Telecomunicaciones, Universidad de las Fuerzas Armadas ESPE, Av. Gral. Rumiñahui s/n, Sangolquí 171103, Ecuador; 2 Research Group of Propagation, Electronic Control, and Networking (PROCONET), Universidad de las Fuerzas Armadas ESPE, Av. Gral. Rumiñahui s/n, Sangolquí 171103, Ecuador; 3 Departamento de Ciencias de la Tierra y la Construcción, Universidad de las Fuerzas Armadas ESPE, Av. Gral. Rumiñahui s/n, Sangolquí 171103, Ecuador; 4 Research Group of Structures and Constructions (GIEC), Universidad de las Fuerzas Armadas ESPE, Av. Gral. Rumiñahui s/n, Sangolquí 171103, Ecuador

**Keywords:** structural health monitoring, wireless network, signal processing methods, accelerometers, earthquakes

## Abstract

Structural health monitoring (SHM) is vital to ensuring the integrity of people and structures during earthquakes, especially considering the catastrophic consequences that could be registered in countries within the Pacific ring of fire, such as Ecuador. This work reviews the technologies, architectures, data processing techniques, damage identification techniques, and challenges in state-of-the-art results with SHM system applications. These studies use several data processing techniques such as the wavelet transform, the fast Fourier transform, the Kalman filter, and different technologies such as the Internet of Things (IoT) and machine learning. The results of this review highlight the effectiveness of systems aiming to be cost-effective and wireless, where sensors based on microelectromechanical systems (MEMS) are standard. However, despite the advancement of technology, these face challenges such as optimization of energy resources, computational resources, and complying with the characteristic of real-time processing.

## 1. Introduction

Ecuador is located in the “Pacific Ring of Fire,” where 85% of the total seismic energy of the planet is released. Therefore, earthquakes with magnitudes greater than six on the Richter scale frequently occur, causing multi-million dollar losses in infrastructure which delays the social and economic development in the affected regions. As an example of a recent event, on 16 April 2016, a 7.8 Mw earthquake struck Ecuador, severely shocking its central coast. In addition to earthquakes, buildings and their structural elements are generally affected by several changes such as deterioration, corrosion, fatigue, chemical reactions, humidity, environmental variables, and dislocations [1]. For these reasons, and to guarantee the operability of a building and the safety of its occupants, it becomes necessary to evaluate the building’s structural behavior through periodic and real-time intelligent monitoring to contrast the current situation with a healthy performance.

Because of the geographical location of Ecuador in the Pacific Ring of Fire, the Nazca Oceanic Plate, and the presence of a complex of local active faults, this country has suffered from severe seismic activity throughout its history [1] which has increased the seismic vulnerability of several buildings. Furthermore, by not considering a correct monitoring system, buildings present serious problems, visible only when the situation is excessively critical, leading, in the worst-case scenario, to the structure demolition due to irreparable damages [2]. One solution to this problem is implementing a network of wireless sensors that allows structural health monitoring (SHM) in buildings [3]. In the event of earthquakes, these SHM systems trigger a warning alert whose data analysis would help experts determine the displacements of the structure and their associated damage limit states. In addition, the system would allow for taking long-term care of the building and reduce the costs associated with repairs and maintenance [4,5,6,7].

The useful life of structures such as buildings, bridges, or factories is directly related to the site’s climatic conditions, structural analysis, and preventive and corrective maintenance. Therefore, these three factors represent vital points when determining if a structure represents a risk for people in the area. Due to the technology advancement, it has become possible to implement monitoring systems at a low cost and extend the useful life of a structure, preventing accidents and premature demolition.

Wireless sensor networks (WSNs) consist of small nodes. Their architecture comprises sensors, controllers, and other devices, allowing communication to collect, process, and transport information to the operator. In its early days, WSNs were created to facilitate military operations. However, their applications have become common in health, traffic analysis, and industrial areas. WSNs consist of one, hundreds, or thousands of sensor nodes. The sensor node equipment usually consists of a radio transceiver with an antenna, a microcontroller, an electronic interface circuit, and a power source, transforming it into a low-power multifunctional device for storing information from environments to perform specific applications [8].

There are certain disadvantages and limitations regarding the physical inspection of structures. For instance, certain places cannot be inspected directly; the need to eliminate materials to check the structure’s state, increased costs associated with the inspection, and an investment of considerable time [9] are all considerable issues. However, seismic structural monitoring systems can identify the damage present in a structure and its level without verifying it visually.

Structural monitoring uses sensors, sensor signal conditioners, data acquisition devices, and hardware and software to interpret the values acquired. The most commonly used devices for measurement are accelerometers, resistance/temperature detectors, thermocouples, strain gauges, corrosion detectors, and linear voltage/displacement transformers. In general, these devices are installed in and attached to the structures. The data they acquire are transmitted through microwaves, radio signals, or fiber optics to the center in charge of interpreting the data where they are stored for later analysis [10].

A building’s dynamic response during an earthquake depends on the relationship between the period of vibration of seismic waves and the structure’s vibration period. When the two periods are equal, this relationship approaches unity, and, consequently, the building enters resonance. If this happens, the acceleration and displacements are greatly amplified, leading to possible structural damage and collapse [11,12].

The analysis of the vibration periods is a parameter of great importance in considering the structural health of buildings since it is a dominant component in the vibrations induced by winds and earthquakes. Analyzing the peak accelerations obtained from the buildings’ dynamic response is necessary to associate it with the damage that may occur at the level of the building components such as equipment or non-structural systems (e.g., ceilings, panels, windows, doors, sanitary installations). Moreover, the velocity analysis is vital to obtaining the abrupt changes between the different times. Sudden changes are associated with dangerous structure responses. Indeed, if a velocity earthquake or pulse earthquake occurs, very high-velocity impulses are registered, which could cause excessive structure damage. Finally, the displacement analysis allows for obtaining the floor drifts. These drifts are the differential or relative shifts between successive levels, computed as the ratio between the relative floor displacement (i.e., the difference between each floor displacement) and the interstory height. Drifts are low or high depending on the structural system of a building. These are valid values to compare the current structure’s performance, the threat it is subjected to, the damage it suffered, and whether it is close to a partial or total collapse.

SHM appeared at the end of the 19th century, at times when people detected faults or cracks in buildings once they heard the acoustic emissions. However, the most used recognition techniques were visual or auditory due to the lack of the necessary technological advance to carry out an adequate study [13]. Fortunately, due to the computational development of the last 30 years, several recognition techniques have been generated based on physical principles and deeper analysis. For example, in recent years, some of the factors monitored in the structure of a building are vibrations, quality of the concrete, inclination, temperature, humidity, the appearance of cracks, fissures, and their alterations [14].

Moreover, SHM consists of using sensors and devices to monitor, analyze, and maintain buildings through techniques used to predict and detect damage early and ensure the safety of structures. In addition, the structure’s velocity and displacement data are calculated, allowing an overall analysis through monitoring. Therefore, several studies focus on designing and analyzing structural monitoring systems. Some study the monitoring of bridges and other buildings [15].

On the one hand, some studies focus on the structural analysis of bridges [16,17]. For instance, the study in [16] proposes an SHM device using three one-axis accelerometers, a microprocessor, an analog-to-digital converter, and a data logger for a long-span bridge. On the other hand, several works focus on analyzing and monitoring buildings [18,19,20]. For instance, the study in [18] proposes the design of a low-cost accelerometer-based wireless sensor node for structural health monitoring applications. This work aims to estimate the system’s performance for permanently monitoring buildings in seismic-prone regions often affected by earthquakes. In addition, this system consists of two wireless sensor nodes with the detection unit onboard and a wireless hub node that acts as the network’s master. The single wireless sensor node prototype is mainly composed of a development board that, through its interfaces, communicates with a radio module, a storage unit, and an acceleration sensor. The results of this study allow the identification of the first three vibration frequencies and the most relevant damping ratios of the structure. Furthermore, the authors in [19] present a system design for measuring the infrastructure’s dynamic acceleration remotely using radio frequency identification tags. This method uses frequency displacement as a primary characteristic for identifying the structure’s state change through piezoelectric accelerometers and data acquisition modules. The results of this study provide information regarding the structure’s natural frequency and make it possible to clearly distinguish and identify changes in the structure state through the measurement of phase shifts in the natural frequency. In addition, the work detailed in [20] analyzes the response of the integrated electronic piezoelectric accelerometer. The results show that the accelerometer responds to small changes in the three axes. Consequently, the system can detect micro-movements in a structure. However, this study does not include experimental results.

Structural health monitoring systems have also significantly increased interest in monitoring cultural heritage structures [21,22,23,24,25,26]. For instance, the studies done in [21] show the application of a WSN for monitoring the structural health of a historic masonry tower. Furthermore, the study presents the generation of mathematical models of the structure based on the information collected by several sensors. The study also shows a custom-made accelerometer with a high-sensitivity piezoelectric accelerometer as a reference. The result of this study shows the way the system used was able to record data with reliability and with no interruptions, providing prevention and risk awareness for the cultural heritage structure. Moreover, [22] presents the application of continuous structural health monitoring (CSHM) on two towers and one sculpture of the medieval era in Italy. The study shows a mathematical algorithm used for an automated operational modal analysis (A-OMA). This algorithm is a modal tracker of dynamic parameters such as main frequencies, mode shapes, and modal damping. With this, the system successfully provided more valuable information for a CSHM. This study also projects their ideas toward the importance of usage and problems with big data in this field. In addition, the study of [24] also shows an interesting application of an SHM on two emblematic historical constructions: a masonry church in Portugal and an adobe church in Perú. This study proposes a methodology for the automatic identification of the structural modal parameters, the process used contains four stages: data acquisition, system identification using the SSI-data method, a cleaning stage of the signal with criteria, and finally, an automatic detection using hierarchical clustering. The results showed an accurate estimation of the modal parameters using just a few sensors and an important influence of the environment over structure’s dynamic properties. Furthermore, the authors in [25] present a method to address the earthquake-induced damage identification on historic towers. The approach proposes the usage of an operational modal analysis (OMA) [27], finite element models (FE) [28], surrogate modeling (SM) [29], and incremental dynamic analysis (IDA) [30]. This study shows the importance of using digital twins created by mathematical modeling of the structure. Furthermore, the results show that SM by itself cannot accurately predict damage to the structure, and IDA analysis may also result in uncertainties if it is used alone. In addition, the predictions can be affected by an error in calibration in most parts. Still, the combined techniques showed successful damage identification, later confirmed by visual inspection. Finally, the study presented in [26] reports a statistical interpretation of structural response parameters performed over 30 years of activity on a steel platform offshore. Although this study represented an essential factor in the management of the platform’s maintenance, it stands out for the use of a correlation between the structure and maritime data, showing consideration of environmental parameters. The structural data collected are an acceleration record of the structure, and the navigational data are the recording of meteorological parameters and behavior of the sea, which is measured by the height of the waves. The results present a structural behavior just like the expected design performed with the structure.

This work aims to collect valuable information that may be useful for future applications of SHM systems in Ecuador or more advanced research because no type of system is currently installed in buildings in the country that allows analyzing its structural state. Furthermore, this analysis identifies the most convenient approaches to contribute to the standardization of SHM systems for post-earthquake structural assessment of strategic and crucial buildings such as hospitals and schools in the country. This would constitute the first step to establishing SHM-based technical procedures for future versions of the Ecuadorian Construction Standard.

## 2. Structural Health Monitoring Systems

### 2.1. Principles

SHM systems comprise the following parts. The first is a network of sensors that collects essential data to determine the structure’s response under seismic activity. Once this data is obtained, it is passed to the next part, which includes a microcontroller or device that receives the data from the sensors. The microcontroller transforms these data into digital information. Then, it sends it to the next part: a data analysis algorithm that processes the signal to acquire characteristics and value parameters. These parameters are used in the last part, constituting the identification and evaluation of damages. This identification is used for generating alarms, notifications, reports of structural health, or even activating an action plan with a separate system. Everything explained is with a general approach as an introduction to SHM systems. Therefore, these parts are deepened in Section 2.2, Section 2.3, Section 2.4 and Section 2.5, exposing their available tools and more characteristics.

The primary trend of these systems shows an approach to a reliable wireless network, such as the systems studied in [31,32,33,34,35]. In addition, SHM systems should be autonomous, efficient, and low-cost, as presented in [36,37,38]. On the other hand, technological progress plays a fundamental role within these systems. For example, in [39,40,41], the Internet of Things (IoT) is used for wireless communication of the system devices, the work presented in [42] uses artificial intelligence for data processing, and the studies presented in [43,44,45] show the use of digital modeling of structures to study their behavior.

### 2.2. Systems Architecture

**Figure 1:**
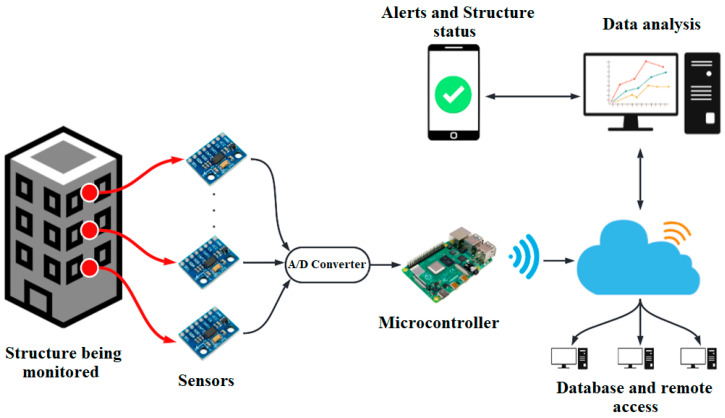
General architecture of SHM systems.

Over the years, various architectures have been proposed and implemented for SHM. However, a general architecture that would be the most complete and could quickly and systematically cover the main components embodied in the various analyzed architectures has been identified. Figure 1 shows a general architecture used for SHM.

The architecture presented in Figure 1 shows a network of sensors (mostly accelerometers) positioned in the structure’s strategic places. They send their information to a digital-analog converter which transmits data to a microcontroller responsible for wirelessly carrying information to a computer through a Wi-Fi module where communication protocols based on the IEEE 802.15.4 standard are used [46]. Once the information reaches the computer, software such as Matlab [47,48] or Python [49] is used to implement an algorithm that aims to process and analyze the data. However, the processing software is of open use when considering the characteristic of a low-cost system. Once the data is processed and interpreted, it is presented either on the computer where the processing is performed or can be sent as alerts and reports to a mobile device to update the user regarding the structure’s status. This information is also sent to cloud storage for later analysis in the field of damage detection or the use of records for simulations of future proposals for new SHM systems. In addition, the advantage of saving the information in the cloud makes it possible to access remote computers so that other users have an idea of the state of the structure or perform an analysis of this data.

This architecture is not fixed because in some studies alarms are not sent to mobile devices since the information is only sent wirelessly for processing. Similarly, in other studies, advanced computer processing is avoided to construct a cost-effective system, and simple processing is performed within the microcontroller. For instance, in [50], the processing is carried out in the microcontroller. If the threshold values are exceeded, a message is sent to a mobile device through a global system for mobile communication (GSM) module to inform the user about the state of the structure.

#### 2.2.1. Sensors, Microcontrollers, and Technologies Used

Within the sensors used in SHM systems, there are several categories where they have microelectromechanical accelerometers (MEMS), piezoelectric accelerometers (PZ) [51], weight sensors, “Fiber Bragg Granting” sensors (FBG) [52], geophones, displacement sensors, humidity sensors, and variations. Specifically, sensors such as ADXL345, ADXL335, ADXL322, Colibrys MS9002, piezoelectric 393B04 PCB, piezoelectric 301A11 PCB, and SDI 1521 have been used.

However, it is evident that in most studies, there is a tendency to use three-axis MEMS accelerometers to acquire vibration data during a seismic movement to process this information and obtain the displacement of a structure [53]. Displacement sensors could indeed be used directly. However, using accelerometers provides low cost, small size, and higher signal quality. In addition, from the accelerometer signal, it is possible to determine several standard value metrics in SHM. It is worth noting that in some interesting works, such as [54], a signal reconstruction technique is used to perform compression of sensor signals with the aim of energy saving and noise reduction. Furthermore, in [55], a three-axis accelerometer is proposed using three one-axis accelerometers to reduce costs while the system demonstrates efficient results.

On the other hand, the most common microcontrollers use Raspberry Pi, as in [56]. Other studies, such as the one presented in [57], use Arduino, whereas the authors in [58] use an FPGA. Once again, the characteristic to be noted in the microcontroller selection is the low cost and efficiency, along with the ability to already have or be able to integrate wireless communication modules and analog–digital converters easily. Similarly, technologies are found, such as using IoT through platforms such as ThingWorx [59], ThingSpeak [60], and Global Positioning System (GPS) synchronization [61] to ensure data integrity.

#### 2.2.2. Sensors Location

The sensor location is a fundamental part of SHM systems. Depending on their location, it is possible to identify the main frequencies optimally. An analysis based on power spectral density (PSD) is used to determine a good location for sensor positioning, as in [62], where accelerometers are positioned in different structure locations. Through analysis with PSD, it is possible to identify the appropriate points to monitor the primary frequencies during an earthquake. The results of this analysis serve to avoid the symmetrical issues of the buildings and demonstrate that the positioning of the sensors must be carried out at critical points such as floor plate joints and plate joints with columns. In addition, it is essential and necessary to place sensors to measure the highest floor of the building [62].

Other interesting findings regarding this topic show the usage of a finite element model to determine the number and placement of sensors in a structure, such as the study presented in [63], where a method to assess this problem is proposed. This method is based on the relation between observed displacements and modal responses using the Fisher information matrix, where a suitable sensor configuration results in the maximization of the norm of this matrix. In addition, in [64], a numerical model is used to assess the optimal sensor positioning and number to ensure the long-term monitoring of the structural health of historic structures. This is accomplished by first obtaining a numerical model based on experimental frequencies. Then, a simulation of ambient vibration is performed where finally, once again, the maximization of the Fisher information matrix is analyzed to obtain the optimal configuration for positioning the sensors and also the number of them. All of these studies have in common the optimization of the number and positioning of sensors which ends up in a low-cost and more dynamic system that obtains valuable information. In addition, something to be noted is the usage of the Fisher information matrix.

### 2.3. Data Processing

In SHM systems, it is common to use the signal from the accelerometers, where the double integration of the acceleration is performed, to obtain the displacement of the structure and to be able to determine its state. Therefore, to begin with, it is essential to filter the signal and then continue interpreting this information to finally execute an action plan that involves alerts, notifications, or activation of some other system, all depending on the obtained data. Therefore, this section focuses on investigating the prosecution and obtaining valuable information.

#### Signal Filtering

Noise filtering in the accelerometer signal is vital since the noise is significantly amplified when performing a double integration. Therefore, even if there is a slight initial noise, there will be a significant noise that noticeably alters the structure displacement signal affecting its interpretation. In this regard, there is high-frequency noise called micro-earthquakes (micro tremors) from 0.5 Hz onwards. There are also low-frequency noises called microearthquakes (microseisms), which are less than 0.1 Hz [65]. Similarly, when integrated twice, the same white noise becomes a phenomenon called “random walk”, which is characteristic of increasing its amplitude over time [66].

**Figure 2:**
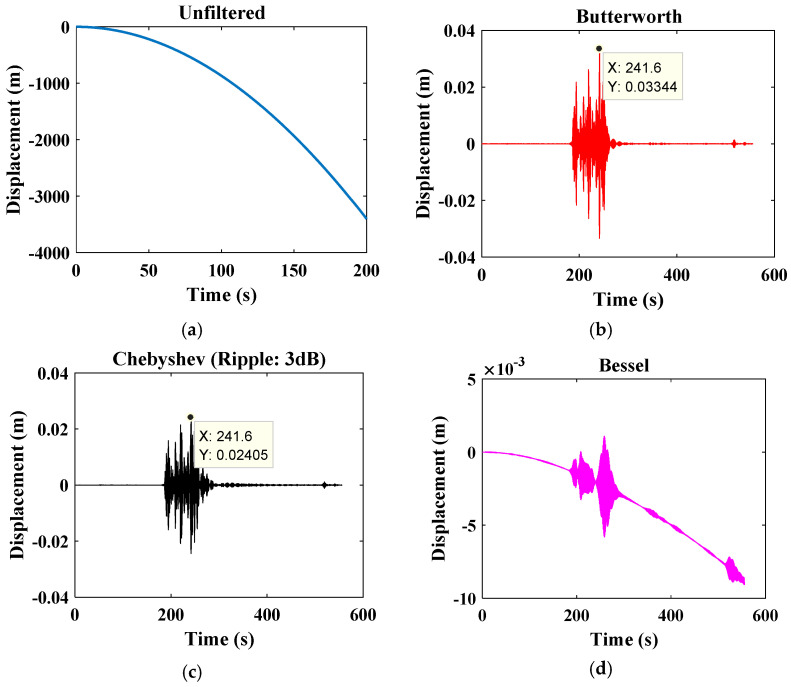
Filtering of the displacement signal (**a**) without filter, (**b**) Butterworth filter, (**c**) Chebyshev filter, and (**d**) Bessel filter.

Baseline or zero correction is vital to eliminating linear trends and low frequency within the accelerometer signal [65]. If the signal is not filtered, it may present characteristics such as those in Figure 2. This graph compares the displacement and time relationship of a signal filtered through different filters and a raw signal without a filter. For instance, the Butterworth filter maintains its shape for higher orders and increases the slope from the cutoff frequency. It has a primarily flat amplitude response. The Butterworth filter has a more linear phase response in the passband than the Chebyshev filters. The amplitude response of the Chebyshev filter has equal amplitude ripples in the passband, and a steeper attenuation slope than Butterworth near the cut. Conversely, the Bessel filter has a nearly linear phase characteristic in the pass region, which provides a primarily flat group delay. It is suitable for pulse circuits because it minimizes hum and overshoot but has a poor attenuation slope. As shown in Figure 2, the Chebyshev filter presents a more pronounced attenuation in the displacement signal. For this reason, the signal amplitude is smaller than the amplitude attenuated by the Butterworth filter. On the other hand, the Bessel filter presents a poor attenuation slope, so the disturbances in the resulting graph continue.

Note that the displacement results from the double integration of an accelerometer signal. Within this example, the way noise can affect the displacement signal is notorious, presenting inadequate values and leading to an erroneous interpretation of the data since the filtered signals suffer from slight movements or almost no displacement while remaining stable. However, when integrated twice, the raw signal increases the amplitude of its noise since these are added as time passes, resulting in a disproportionate displacement signal compared to the results obtained with the filtered signals. Filters such as Butterworth, Chebyshev, Bessel, Ormsby, Bartlett, Hanning, and finite impulse response [67] are usually used to eliminate this noise.

### 2.4. Data Processing Techniques

Several signal processing techniques in SHM solve noise and disturbance problems in the signal. The most widely used methods are the wavelet transform (WT), Kalman filter (KF), fast Fourier transform (FFT), time series statistical models (TS), and Hilbert–Huang transform (HHT). The WT is mainly used to remove noise and detect damage to structures. In addition, it is common to use the FFT and WT to obtain the frequency spectrum of the accelerometers and help design filters. Table 1 presents an analysis of the advantages and disadvantages of the different processing techniques commonly used in SHM systems:

### 2.5. Damage Identification and Assessment Techniques

Mainly studies such as [76,77] present algorithms based on artificial intelligence to identify and evaluate damages. The rapid increase in research with machine learning (ML) applications suggests that due to the emergence of technologies such as the IoT and the handling of large volumes of data, it is necessary to use techniques that guarantee an accurate and rapid response regarding the damage identification and evaluation. In this regard, the most used methods in the literature are decision trees, ML, deep learning, Bayesian classifiers, fuzzy logic, Gaussian mixture, support vector machines, hidden Markov model and neural networks, and their variations such as multilayer neural networks, probabilistic neural networks, a neural network with backpropagation, and convolutional neural network, among others. In addition, other algorithms that involve statistical analysis and the use of probabilities are also used, such as the one presented in [78], based on Bayesian probability.

The algorithms use information from records of previously occurred earthquakes for their training. Similarly, they use digital and mathematical modeling of structures with characteristic information from other sensors such as humidity, crack, ultrasonic, and temperature. This way, the focus is managing valuable information for an adequate diagnosis. Then, these algorithms are evaluated through artificially induced vibrations or movements that simulate an earthquake [79].

As a result, the algorithms for identifying and assessing damages in SHM demonstrate exceptional capacity and precision. However, the computational cost is also considered for autonomous and low-cost systems. Therefore, some studies aim to optimize the computational burden [80].

### 2.6. Challenges for SHM Systems

As SHM systems advance, challenges must be addressed for more autonomous, sophisticated, and cost-efficient systems. The SHM challenges to be overcome are the following:

- Autonomy of the accelerometers with less power consumption is needed in the order of mA at 3 V.
- Accelerometers must have high sensitivity, data transmission, and sampling speed.
- Within the information analysis, algorithms can reveal possible damages in the structures based on the recorded information.
- Wireless systems.
- Real-time data processing.
- An automated procedure for operational modal analysis (A-OMA) for large datasets used in long-term monitoring systems [81].

In this regard, several studies have been developed in recent years. Table 2 summarizes the main characteristics and results of different analyzed works.

**Table 2:** Summary of the main characteristics and results of the state-of-the-art SHM works.

Reference	Data Processing	Sensors	Technologies	Results
[7]	•Tensor completion method	•Vibration sensor	-	•Cost-effective •Fast and efficient damage assessment
[9]	•Convolutional neural network based on the structure’s 3D generated model	-	•Neural network	•Fast and efficient security assessment
[14]	-	•ADXL345 •Piezoelectric accelerometer •FBG sensors •Ultrasonic •Pressure sensors •Crack sensor	•IoT	•The integration of several sensors increases the accuracy of structural monitoring •Using IoT devices facilitates the communication of information
[18]	-	•FBG sensors •MEMS	-	•Sending danger notifications to users •Low-cost system •Good performance
[31]	-	•Load cell •MEMS	•Wireless (ZigBee)	•A non-intrusive system without efficient wiring
[32]	•Two algorithms are used: (i) The first algorithm considers the variation in the measurements of the different sensor nodes; (ii) the second algorithm focuses on fault detection and sensor data collection based on a historical calibration basis	•Accelerometer 393B04PCB •Piezoelectric sensors	•Wireless (ZigBee)	•A system that uses the combination of two types of algorithms to identify threshold violations
[33]	•Network flooding algorithm used for efficient data communication	**-**	•OMNET++	•Good algorithm performance compared to standard communication protocols
[34]	-	-	•Raspberry pi •CC3200 Wi-Fi	•Efficient information synchronization algorithm •Real-time •Cost-effective •Low power consumption
[35]	•netSHM (Algorithm created for the identification of damage in structures)	-	•Wireless	•Identifying significant changes in the structure stiffness •Induced damage identification •A robust damage identification algorithm
[36]	-	•FBG sensors	-	•Sending alerts to users •Highly efficient automated system •Use of cost-effective materials that take up little space
[37]	•Bayesian theory	•Piezoelectric accelerometer •MEMS •FBG	•Matlab •OpenSees	•Low-cost system •Real-time monitoring •Efficient results using Bayesian theory
[38]	-	•Fiber-optic sensor	•Wireless	•Low-cost wireless sensors •Approach to detect structures affected by corrosion
[39]	-	•Accelerometers	•IoT	•Efficient bridge’s SHM •Good performance in IoT communication
[40]	-	•MEMS ADXL 355	•IoT •STATOTEST (sensor developed)	•Recording minimal inclinations with great precision •Low-cost system
[41]	-	•Triaxial MMA8452Q accelerometer •ADXL362 accelerometer	•ATMEGA 328 •CC3000 Wi-Fi •Xnode board •ESP 8266 •IoT	•Presenting a review of future SHM systems •Analyzes several sensors and detection algorithms
[42]	Computes three metrics: cumulative absolute velocity (CAV), relative CAV, and total CAV deviation, used for damage assessment	-	•Machine learning	•The ORL machine learning model shows an identification accuracy of 93% to 97.5%
[43]	-	-	•Building information model (BIM)	•Developing the digital model of a structure •Efficient structure behavior analysis
[44]	•Fast Fourier transform • Bayesian probability	-	•Generation of 3D models	•Increase in vibration identification by using the 3D model •Accuracy in damage detection based on Bayesian probability and 3D model
[45]	•Fuzzy neural network •ANN-type multilayer feedforward •Multi-Stage ANN •Probabilistic ANN •Bayesian decision tree	-	•machine learning •ANN	•It presents several methods for identifying structural damage using machine learning, artificial intelligence, and deep learning
[46]	-	•MEMS Colibrys •MS9002	•IEEE 802.15.4 •GPS •Kinetis KL15 •NEO6-M Xbee module •Wireless	•Perfect data synchronization at a sample rate of 1000 Hz •Possibility of having several nodes with wireless communication sensors
[47]	•Runge–Kutta method •Logical analysis of data (LAD)	-	•Matlab	•The LAD model provides an efficient technique to learn, simulate, and predict the structure behavior dynamic response
[48]	•Wavelet transform	-	•ASCE benchmark •Matlab	•Efficiently detects sudden changes in structure damage •Provides increased damage information compared to traditional methods
[49]	•Fast Fourier transform	•ADXL322 accelerometer •393B12 accelerometer	•MSP430F538a controller • IEEE 802.15.4 standard •Python	•Accurate fault detection in buildings
[50]	-	•ADXL335 triaxial sensors	•GSM	•Damage detection with an unprecedented level of severity •Non-intrusive system •Sending alerts to users •Low-cost system •Low-energy consumption system
[51]	•Enhanced frequency domain decomposition method	•Biaxial MEMS •PCB/393B12 and PCB/393B31 piezoelectric sensors	-	•Relatively efficient SHM •Vibration signals are mistaken for noise when using MEMS
[52]	•Current-voltage curves interpretation	•FBG	-	•Allowing structural damage detection and evaluation by analyzing the sensor’s ohmic behavior curve
[53]	•Structural Analysis and design Vi8 Pro (finite element calculation program used for structural analysis in buildings, plants, and other structures)	•ADXL345 accelerometer	•STAAD Vi8 Pro	•Effective information synchronization and analysis
[54]	•Signal reconstruction using complex algorithms	-	•Compressed sensing technique	•Energy saving •The vibration signals can be compressed to a large extent without intruding on the quality of the reconstructed structural parameters when the Peak SNR remains above 20 dB
[55]	•Design of a cost-effective three-axis accelerometer for SHM	•SDI 1521 accelerometer •PCB 301A11 accelerometer	•ADS1258 A/D converter • Data logger and internet connection for remote monitoring and diagnostic access	•Effective operation •Minimization of costs by 64.3% compared to other systems
[56]	•Fast Fourier transform •Subwoofer method for calibration	•ADXL345 accelerometer	•Raspberry Pi 3	•The subwoofer method effectively calibrates the accelerometer •Presents measurements with an error of 3.65%
[57]	-	•Geophone	•Arduino Uno	•Efficient system •Low-cost system •Low-energy consumption system
[58]	•Vertical and horizontal vibration computation method	•FBG sensor arrays	•FPGA •JAVA Cosmos	• Real-time vibration monitoring •Sending alerts to users •Sensor location analysis
[59]	•Fast Fourier transform •Wavelet transform •Cross correlation •Orthogonal Transform	•Piezoelectric sensors	•IoT • Raspberry Pi controller with MCP3008 A/D module and Wi-Fi module •ThingWorx for cloud data storage •Wireless	•Sending notification of danger to the user •Information recorded in the cloud •Remote monitoring
[60]	-	•ADXL345 accelerometer •BF350 3AA pressure sensor •Humidity sensor	•ThingSpeak •IoT •Matlab •C# •Wireless	•Cost-efficient •An early solution to structures at risk of collapse
[61]	•Standing wave method	•A-1637 accelerometer	•GPS synchronization	•Determining the dynamic state of buildings and structures based on microseismic vibrations
[62]	•Power spectral density	•24-bit Reftek accelerometers model 130 SMA	-	•Low-energy consumption system •Key points such as floor plate joints and joints between floors and columns were determined
[82]	• Mark, Class, Time sampling process	•Smart transducers	•IEEE1451 standard	•Detecting the arrival of a destructive earthquake in real-time •Broadcasting a warning signal
[65]	•Baseline correction •Butterworth filter •Chebyshev filter •Riffle filter •Bessel filter	•MEMS	• Standard energy efficiency data	• Analysis of various filters for data processing
[66]	•Fast Fourier transform •Auto-correlation functions •Time-varying spectral analysis techniques	-	•Matlab •REC_MIDS toolbox	•Sending notification of danger to the user •Real-time system identification and damage detection •Allows estimating of modal displacements at non-instrumented floors
[67]	•Fast Fourier transform •Infinite impulse response filtering algorithm	-	-	•Elimination of noise and intrusive component frequencies using IIR filters •Dynamic identification of the natural frequencies
[68]	•Power spectral density •Frequency domain decomposition (FDD) •Stochastic subspace identification •Fast Fourier transform •Peak-picking •Eigen-system realization algorithm •Blind source separation •Empirical mode decomposition •Singular value decomposition	•Piezoelectric sensors •Triaxial MEMS	•Matlab •ARTeMIS software •MACEC software •PULSE software •IoT	•Trend of wireless SHM systems •Highlights data handling techniques such as FDD and SSI
[69]	•Wavelet transform •Statistical models •Hilbert–Huang transform •Fast Fourier transform •Cohen’s class •Kalman filter •S transform •Short FFT	-	-	•Highlights the wavelet transform and the Hilbert–Huang transform to remove signal noise and detect damage to structures
[70]	•Unscented Kalman filter	-	•OpenSees software	•Identifies the properties of the structure with different levels of elasticity and seismic loading in a building
[72]	•Extended Kalman filter	-	•Estimation of ground movement with digital techniques	•Provides satisfactory ground motion estimation under realistic levels of measurement noise and partial measurements
[73]	•Fast Fourier transform •ANN	•Accelerometers	•ASCE Benchmark	•High-precision identification algorithm for frequency detection
[74]	•Synchronized wavelet transform	-	-	•The synchronized wavelet transform outperforms damage detection compared to other methods, obtaining a minimum error of 0.12%
[75]	•Fast Fourier transform	•MEMS	-	•An efficient noise removal system •Efficient SHM of bridges
[76]	•Decision trees •Neural network •Ensemble methods •Support vector machines •Back propagation neural network •K-nearest neighbors •Gaussian mixture •Hidden Markov model	-	•Machine learning	•Highlights the increase in Machine Learning-based studies for SHM
[77]	•Fuzzy neural network •Multilayer feedforward ANN •Multi-stage ANN •Probabilistic ANN •Bayesian decision tree	-	•Machine learning •ANN	•Presents various methods of identifying damage to structures using machine learning, artificial intelligence, and deep learning
[78]	•Transfer Bayesian learning	-	-	•Allows a probabilistic identification of damage to the structure
[79]	•Singular spectrum analysis (SSA)	-	-	•SSA is a non-parametric spectral estimation method •Enables efficient damage assessment after earthquakes
[80]	•Convolutional neural network	•TROMINO accelerometrer	•MS Visual Studio C++ •Matlab •Wireless	•Wireless and decentralized SHM system •Low-cost system •Computational cost optimization

## 3. The Usage of SHM Systems in Ecuador

Ecuador is characterized by its high seismicity and critical economic losses and death toll after severe earthquakes [1,83] that have forced the authorities to keep strengthening the construction standards through advanced theories and technologies. Indeed, since the beginning of 2021, several chapters of the structural area from the Ecuadorian Construction Standard have undergone improvements whose evidence will be reflected in 2023. One of these new approaches is to include a section in the Seismic Risk, Evaluation, and Structural Retrofit Chapter [84] regarding the regularization of SHM systems, especially for critical buildings such as schools, hospitals, and emergency response infrastructure. The primary purpose is to guide the construction professionals on how, when, and where to install different sensors and distinguish the various responses from the buildings to alert the users or officials and act accordingly to the warning message.

**Figure 3:**
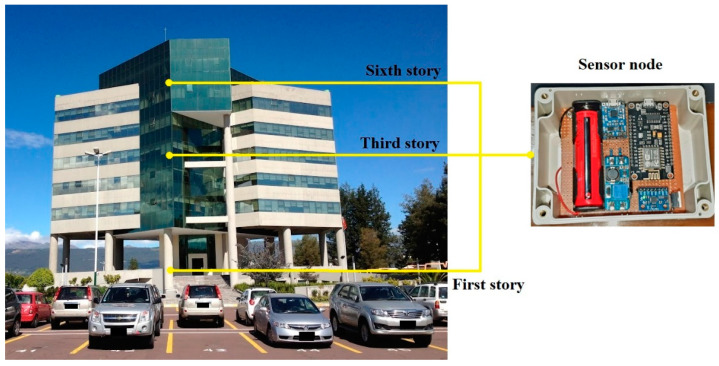
Location of the sensor nodes in the main administrative building of Universidad de las Fuerzas Armadas ESPE, Sangolquí, Ecuador.

In this context, a research project has been proposed at Universidad de las Fuerzas Armadas ESPE in the mountain region of Ecuador, characterized by its high seismicity. The first stage of this project aims to develop a system capable of collecting vibration information through a network of sensors located on the first, third, and sixth stories in the university’s main administrative building; see Figure 3. The sensor node comprises a three-axial accelerometer in charge of collecting information in the building and a development board which is used to obtain and transmit data through the MQTT protocol to a broker-server.

**Figure 4:**
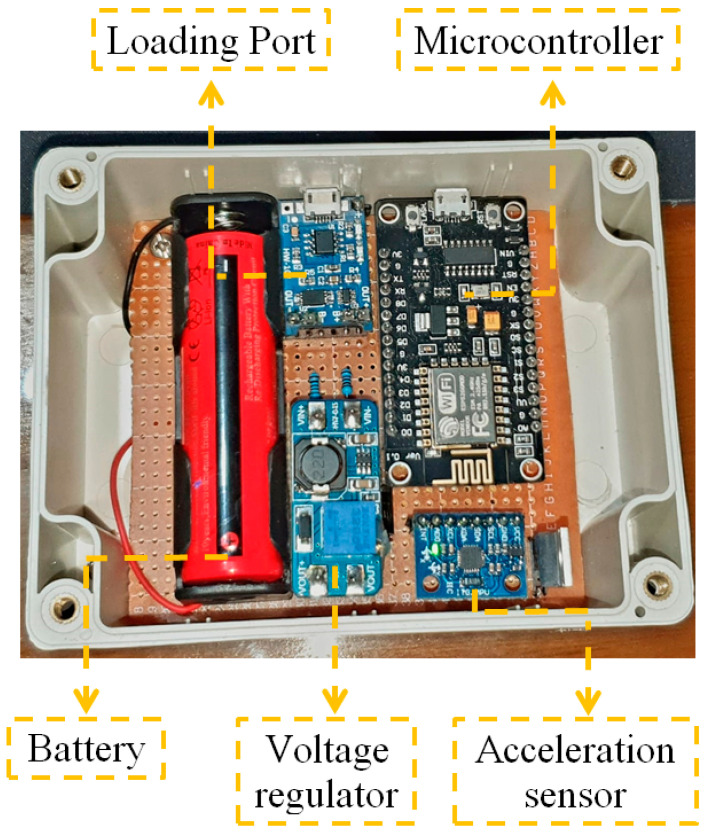
Sensor node structure and components.

In detail, the sensor node comprises a three-axial MPU6050 accelerometer with MEMS (micro-electro-mechanical systems) technology, a micro-machined structure built on a silicon wafer. In addition, it features a NodeMCU v3 ESP8266 microcontroller, an Arduino-compatible WiFi development module usually used for IoT applications. It presents recharging and power circuits made up of a Tp4056 charger with overload, over-discharge, and short-circuit protection for batteries, followed by an MT3608 voltage regulator for a constant voltage supply to the microcontroller. In addition, the sensor node has a 6800 mAh 18,650 rechargeable battery to keep it working in case the main power supply is interrupted, as shown in Figure 4.

Furthermore, the server has an application divided into two sections: Acquisition and processing.

**Figure 5:**
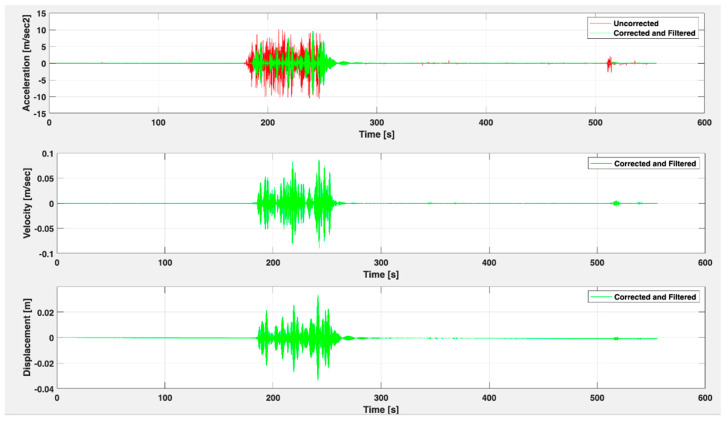
Acceleration, velocity, and displacement display window with baseline correction and filtering.

The acquisition software interface has four windows. The first three windows allow real-time monitoring of the three three-axial sensor nodes. It has a cursor tool for each axis of the sensor node. It has an alarm window, which provides real-time data history detailing the sensor, axis, value, and date/time that exceeded the offset established by the user. The processing software interface will report corrected data for the accelerations and calculated velocities and displacements. Indeed, the software has a window with correction tools. The baseline correction seeks to correct the distortion generated by the digitization of the signal and the introduction of low-frequency components, which create non-null or oscillatory resulting graphs around zero. Filter correction allows applying a Butterworth filter of the high-pass, low-pass, or band-pass type as the user deems necessary. Filtering removes noise caused by external equipment or ambient noise in the environment. By applying the tools, the resulting acceleration, velocity, and displacement graphs will be similar to those in Figure 5.

**Figure 6:**
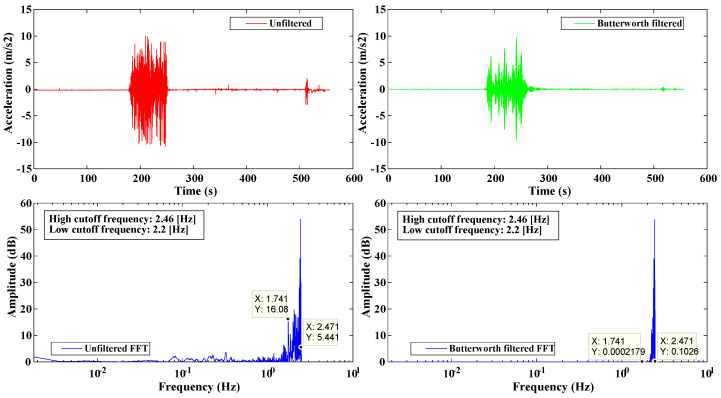
Fast Fourier spectrum of unfiltered and Butterworth filtered acceleration signals.

In addition, the software allows obtaining the Fourier transform of the acceleration signal, which is used to determine the sensor oscillation frequency; see Figure 6. Finally, another window will show the wavelet transform, a power graph as a function of frequency and time used to analyze the frequency content and released energy contained in the earthquake records. During an important earthquake, the processed data will trigger warning signals if the seismic building response exceeds standardized engineering parameters such as interstory drifts to take immediate action. Further experience will contribute to standardizing the implementation and usage of SHM systems in strategic buildings and consequently increase the available data to improve the Ecuadorian Construction Standard.

## 4. Discussion and Conclusions

This work has reviewed technologies, architectures, data processing techniques, identification techniques, and challenges faced in SHM systems. In addition, this work has distinctively approached collection of information that may be useful for future standardized applications of SHM systems in Ecuador or more advanced research on these issues.

It should be noted that the advancement of technology has dramatically benefited SHM systems, providing architectures that especially involve the IoT for wireless measurement and communication devices. These, in turn, send information to the cloud or a database, which characterizes the use of large volumes of information that are used for real-time diagnosis or the evaluation of damage and records that are of value for future experiences that include the use of historical data. These systems, having the characteristic of a wireless communication network, significantly exceed the classic designs that use wiring, both in data collection and in their cost, due to the need to have reliable communication that does not collapse along with the structures they are to monitor.

Essentially, in this work, it has been identified that SHM systems comprise a data acquisition system or measurement system through sensors located in the structure’s strategic positions that are being monitored and a data processing algorithm that filters and interprets the information the sensors acquired to initiate action plans such as sending notifications or alarms to users to avoid more significant damage that the earthquake may cause. Similarly, the feature to note is the cloud information storage with which an evaluation of the state of health and identification of damage to the structure can be carried out. This work has presented an architecture that can be considered the most complete by adopting the best characteristics of several analyzed architectures.

The technological approach presented in the reviewed studies points to cost-efficient and autonomous systems which should have lower energy consumption but without reducing monitoring quality. Most research has used MEMS accelerometers to fulfill this characteristic where specifically the ADXL345 sensor stands out for being commonly used in SHM applications. However, novel technologies such as FBG and piezoelectric sensors have also shown exciting solutions.

Since this review has presented several technologies, it is necessary to point out their advantages and disadvantages. The usage of technologies that ensure a low-cost system can increment the accessibility to them. Technology such as a three-axis accelerometer using three one-axis accelerometers can benefit this purpose, overcoming the classical wired systems. However, problems such as data synchronization and the sensor precision can be a drawback. In addition, the algorithms used for synchronization can increase the computational cost as they would need a certain complexity to overcome the standard systems. In addition, wireless accelerometers present several weaknesses such as the synchronization of the signals to extract the mode shapes, and commonly the energy consumption of these devices is another limitation.

On the other hand, using methods to determine the number and positioning of sensors can significantly help optimize the system, providing a low-cost and more dynamic approach. Still, this drawback is the uncertainties that arise with experimental testing which can complicate the process. These uncertainties have to be noted to obtain a long-term and reliable system. However, artificial intelligence or machine learning in these systems has shown an improvement in the identification and damage assessment field. An apparent limitation of this technology could be that it involves high computational processing, which could be solved using cloud computing. However, it has recently been demonstrated that tiny machine learning (TinyML) solutions, characterized by low computational effort and memory/time consumption since the inference is made on embedded systems, could be effective for vibration-based SHM.

In addition, even though the requirements of heavy computational processing can be done all in the cloud, saving space and money, this kind of system stills has a drawback since working on the cloud means that the data can be compromised or modified if it is not well guarded with cybersecurity.

On the other hand, filtering the accelerometer signal is necessary for eliminating both high- and low-frequency noise. In addition, the baseline correction is vital since when analyzing the signal, if it is not filtered, results can lead to an erroneous interpretation and, therefore, inadequate monitoring of the structural health status. Consequently, it has been found that filters and data processing methods are required. The standard processing techniques from the literature for SHM applications are the wavelet transform, the fast Fourier transform, the Kalman filter (KF), and combinations of these methods with more sophisticated techniques where statistical analysis is available to remove noise and polish the signal for proper interpretation. In the same way, the identification and detection of damage in a structure have involved methods with machine learning and artificial intelligence in general, where an overview of the optimization of these algorithms shows to save computational consumption.

Moreover, the applications of SHM systems present a future with several limitations and challenges to overcome, such as minimum energy consumption, higher precision sensors, and greater accuracy in damage identification algorithms with less computational consumption. Research development and technological advancement in this field collaborate to mitigate these limitations, which contribute to the optimization and improvement which means an increase in the safety of people facing the devastating consequences and effects of an earthquake.

Finally, this work has presented the first attempt at an SHM system in Ecuador. This system acquires acceleration data in the first, third, and sixth stories of a building through a sensor network. The SHM system prototype comprises three sensor nodes with an MPU6050 three-axial accelerometer with capacitive MEMS technology, a NodeMCU v3 ESP8266 microcontroller developed with WiFi, and a power and recharge circuits for a 6800 mAh 18,650 rechargeable battery. The sensor node transmits the collected data using the MQTT protocol. In addition, the software has been developed for real-time monitoring of the acceleration transmitted by the sensor node and processing of the collected data, which will be used to determine engineering demand parameters to trigger alerts in the case of an earthquake.

## Figures and Tables

**Table 1:** Common processing techniques in SHM systems [68,69,70,71,72,73,74,75].

Technique	Description	Advantages	Drawbacks
Time series statistical models (TS)	They are used to develop an approximate mathematical model based on input and output measurements.	•Easy to implement •Different models to be used	•Noise sensitive •Used to model linear systems
Wavelet transform (WT)	The WT provides a time-frequency signal representation through the scale and time window function.	•Good resolution in the time-frequency domain •Good signal-to-noise ratio •It has a large selection of Wavelet models	•Spectral fugue •Requires various levels of decomposition •The selection of the mother wavelet can affect the results
Wiener filter	It uses statistical methods to approximate the signal to one without noise. It is characteristic of being a time-invariant filter.	•It considers the statistical noise behavior	•Linear behavior
Hilbert–Huang transform (HHT)	It is based on two steps: an empirical mode decomposition followed by the Hilbert spectral transform (HT).	•Adaptive method •Easy to implement •Good resolution in the time and frequency domain	•Requires calibration
Fast Fourier transform (FFT)	The FFT converts discrete samples of a continuous time series signal to a frequency domain representation.	•Can model linear and nonlinear systems •Easy to implement •Simplicity •Computationally efficient	•It is inefficient in complex systems •Requires calibration to find model order •Noise sensitive •It only has frequency representation •Its resolution depends on the number of samples
Short-time Fourier transform (STFT)	It is an extension of the FFT capable of analyzing non-stationary signals. The STFT can represent the variation of the signal’s frequency content as the signal changes in time by dividing the signal into small time windows where each window is analyzed using the FFT.	•Easy to implement •Time-frequency representation •Simplicity	•Limited time/frequency resolution •Its resolution depends on the number of samples. •Nonlinear signals cannot be adequately analyzed
Bilinear time-frequency distributions (Cohen’s class)	It is a method to estimate the energy of time-varying systems.	•Computationally efficient •High resolution in the time-frequency domain	•It is not adaptive •Large computational processing time
Kalman filter (KF)	It is an optimal algorithm for recursive data processing capable of estimating the linear dynamic system.	•Good signal-to-noise ratio •It presents a reasonable estimation of the rate of change over time	•Requires calibration parameters •Large computational processing time •Limited tracking accuracy •Nonlinear systems can use only one version of the algorithm
S transform	It is a time-frequency distribution that combines ideas from WT and a scalable, moving Gaussian location window to adapt the time resolution depending on the signal’s frequency content.	•Good resolution in the time and frequency domain •Spectrum components can be located in the time domain	•Requires calibration •Large computational processing time •It is not adaptive
Blind source separation (BSS)	The BSS is capable of revealing mixed features in the measured data.	•Good signal-to-noise ratio •Good precision in separating the frequency components	•Requires calibration • Nonlinear and transient signals cannot be adequately analyzed

## References

[1] Lanning F., Haro A.G., Liu M.K., Monzón A., Monzón-Despang H., Schultz A., Tola A. (2016). EERI Earthquake Reconnaissance Team Report: M7.8 Muisne, Ecuador Earthquake on April 16, 2016.

[2] Shabani A., Alinejad A., Teymouri M., Costa A.N., Shabani M., Kioumarsi M. (2021). Seismic Vulnerability Assessment and Strengthening of Heritage Timber Buildings: A Review. Buildings.

[3] Giurgiutiu V. (2008). Structural Health Monitoring.

[4] Yeow T.Z., Kusunoki K. (2022). Unbiased rank selection for automatic hysteretic response extraction of RC frame buildings using acceleration recordings for post-earthquake safety evaluations. Earthq. Eng. Struct. Dyn..

[5] Komec Mutlu A., Tugsal U.M., Dindar A.A. (2022). Utilizing an Arduino-Based Accelerometer in Civil Engineering Applications in Undergraduate Education. Seismol. Res. Lett..

[6] Cardoni A., Borlera S.L., Malandrino F., Cimellaro G.P. (2022). Seismic vulnerability and resilience assessment of urban telecommunication networks. Sustain. Cities Soc..

[7] Lin J.-F., Li X.-Y., Wang J., Wang L.-X., Hu X.-X., Liu J.-X. (2021). Study of Building Safety Monitoring by Using Cost-Effective MEMS Accelerometers for Rapid After-Earthquake Assessment with Missing Data. Sensors.

[8] Sabato A., Niezrecki C., Fortino G. (2017). Wireless MEMS-Based Accelerometer Sensor Boards for Structural Vibration Monitoring: A Review. IEEE Sens. J..

[9] Tsuchimoto K., Narazaki Y., Hoskere V., Spencer B.F. (2021). Rapid postearthquake safety evaluation of buildings using sparse acceleration measurements. Struct. Health Monit..

[10] Davis A.M., Mirsayar M., Hartl D.J. (2021). A novel structural health monitoring approach in concrete structures using embedded magnetic shape memory alloy components. Constr. Build. Mater..

[11] Marasco S., Cimellaro G.P. (2021). A new evolutionary polynomial regression technique to assess the fundamental periods of irregular buildings. Earthq. Eng. Struct. Dyn..

[12] Chieffo N., Formisano A., Mochi G., Mosoarca M. (2021). Seismic Vulnerability Assessment and Simplified Empirical Formulation for Predicting the Vibration Periods of Structural Units in Aggregate Configuration. Geosciences.

[13] Gopinath V.K., Ramadoss R. (2021). Review on structural health monitoring for restoration of heritage buildings. Mater. Today Proc..

[14] Mishra M., Lourenço P.B., Ramana G.V. (2022). Structural health monitoring of civil engineering structures by using the internet of things: A review. J. Build. Eng..

[15] Sofi A., Jane Regita J., Rane B., Lau H.H. (2022). Structural health monitoring using wireless smart sensor network—An overview. Mech. Syst. Signal Process..

[16] Lin C.-H., Chen S.-Y., Yang C.-C., Wu C.-M., Huang C.-M., Kuo C.-T., Huang Y.-D. Structural health monitoring of bridges using cost-effective 1-axis accelerometers. Proceedings of the IEEE Sensors Applications Symposium.

[17] Lin C.-H., Chen S.-Y., Kuo C.-T., Sung G.-N., Yang C.-C., Wu C.-M., Huang C.M. A real-time bridge structural health monitoring device using cost-effective one-axis accelerometers. Proceedings of the IEEE Tenth International Conference on Intelligent Sensors, Sensor Networks and Information Processing.

[18] Valenti S., Conti M., Pierleoni P., Zappelli L., Belli A., Gara F., Carbonari S., Regni M. A low cost wireless sensor node for building monitoring. Proceedings of the IEEE Workshop on Environmental, Energy, and Structural Monitoring Systems.

[19] Jayawardana D., Kharkovsky S., Liyanapathirana R., Zhu X. (2016). Measurement System with Accelerometer Integrated RFID Tag for Infrastructure Health Monitoring. IEEE Trans. Instrum. Meas..

[20] Abdullahi S.I., Che Mustapha N.A., Habaebi M.H., Islam M.R. (2019). Accelerometer Based Structural Health Monitoring System on the Go: Developing Monitoring Systems with NI LabVIEW. Int. J. Online Biomed. Eng..

[21] Barsocchi P., Bartoli G., Betti M., Girardi M., Mammolito S., Pellegrini D., Zini G. (2021). Wireless Sensor Networks for Continuous Structural Health Monitoring of Historic Masonry Towers. Int. J. Archit. Herit..

[22] Zini G., Bartoli G., Betti M., Marafini F. A quality-based framework for data-driven SHM of heritage buildings. Proceedings of the IEEE Workshop on Complexity in Engineering (COMPENG).

[23] Bartoli G., Betti M., Girardi M., Padovani C., Pellegrini D., Zini G. (2022). Dynamic monitoring of a tunnel-like masonry structure using wireless sensor networks. Proceedings of the Institution of Civil Engineers—Structures and Buildings.

[24] Zonno G., Aguilar R., Boroschek R., Lourenço P.B. (2018). Automated long-term dynamic monitoring using hierarchical clustering and adaptive modal tracking: Validation and applications. J. Civ. Struct. Health Monit..

[25] Kita A., Cavalagli N., Venanzi I., Ubertini F. (2021). A new method for earthquake-induced damage identification in historic masonry towers combining OMA and IDA. Bull. Earthq. Eng..

[26] Betti M., Castelli P., Galano L., Spadaccini O., Zini G. (2022). Long-Term Structural Monitoring of a Steel Jacket Offshore Platform. Validation of Meteo-Marine Data and Implications for Maintenance. European Workshop on Structural Health Monitoring.

[27] Brincker R., Ventura C.E. (2015). Introduction to Operational Modal Analysis.

[28] Kotsovos M.D. (2015). Finite-Element Modelling of Structural Concrete.

[29] Forrester A.I.J., Sóbester A., Keane A.J. (2008). Engineering Design via Surrogate Modelling.

[30] Vamvatsikos D., Cornell C.A. (2002). Incremental dynamic analysis. Earthq. Eng. Struct. Dyn..

[31] Sindhuja S., Kevildon J.S.J. MEMS-based wireless sensors network system for post-seismic tremor harm evaluation and building monitoring. Proceedings of the International Conference Circuits, Power Computing Technologies.

[32] Morello R., De Capua C., Meduri A. Remote monitoring of building structural integrity by a smart wireless sensor network. Proceedings of the IEEE Instrumentation & Measurement Technology Conference Proceedings.

[33] Muñoz J., González R., Otero A., Gazca L., Huerta M., Sagbay G. A flooding routing algorithm for a wireless sensor network for seismic events. Proceedings of the International Conference on Computing Systems and Telematics.

[34] Jornet-Monteverde J.A., Galiana-Merino J.J., Soler-Llorens J.L. (2021). Design and Implementation of a Wireless Sensor Network for Seismic Monitoring of Buildings. Sensors.

[35] Chintalapudi K., Fu T., Paek J., Kothari N., Rangwala S., Caffrey J., Govindan R., Johnson E., Masri S. (2006). Monitoring civil structures with a wireless sensor network. IEEE Internet Comput..

[36] Wu D., Peng B., Xu Q. A building structure health monitoring system based on the characteristic of TFBG. Proceedings of the 9th International Conference on Optical Communications and Networks (ICOCN 2010).

[37] Sivasuriyan A., Vijayan D.S., Górski W., Wodzyński Ł., Vaverková M.D., Koda E. (2021). Practical Implementation of Structural Health Monitoring in Multi-Story Buildings. Buildings.

[38] Riggio M., Dilmaghani M. (2020). Structural health monitoring of timber buildings: A literature survey. Build. Res. Inf..

[39] Iasha F., Darwito P.A. Design of Algorithm Control For Monitoring System And Control Bridge Based Internet of Things (IoT). Proceedings of the International Conference on Smart Technology and Applications.

[40] Balek J., Klokočník P. (2021). Development of low-cost inclination sensor based on MEMS accelerometers. IOP Conf. Ser. Earth Environ. Sci..

[41] Basko A., Ponomarova O., Prokopchuk Y. (2021). Review of Technologies for Automatic Health Monitoring of Structures and Buildings. Int. J. Progn. Health Manag..

[42] Muin S., Mosalam K.M. (2021). Structural Health Monitoring Using Machine Learning and Cumulative Absolute Velocity Features. Appl. Sci..

[43] Theiler M., Dragos K., Smarsly K. BIM-based Design of Structural Health Monitoring Systems. Proceedings of the Structural Health Monitoring.

[44] Li Z., Hou J., Jankowski Ł. (2022). Structural damage identification based on estimated additional virtual masses and Bayesian theory. Struct. Multidiscip. Optim..

[45] Lei Y., Rao Y., Wu J., Lin C.-H. (2020). BIM based cyber-physical systems for intelligent disaster prevention. J. Ind. Inf. Integr..

[46] Giammarini M., Isidori D., Concettoni E., Cristalli C., Fioravanti M., Pieralisi M. Design of Wireless Sensor Network for Real-Time Structural Health Monitoring. Proceedings of the IEEE 18th International Symposium on Design and Diagnostics of Electronic Circuits & Systems.

[47] Abd-Elhamed A., Shaban Y., Mahmoud S. (2018). Predicting Dynamic Response of Structures under Earthquake Loads Using Logical Analysis of Data. Buildings.

[48] Hera A., Hou Z. (2004). Application of Wavelet Approach for ASCE Structural Health Monitoring Benchmark Studies. J. Eng. Mech..

[49] Raju K.S., Sahni Y., Pratap Y., Naresh Babu M. Implementation of a WSN system towards SHM of civil building structures. Proceedings of the IEEE 9th International Conference on Intelligent Systems and Control.

[50] Sivagami A., Jayakumar S., Kandavalli M.A. Structural health monitoring using smart sensors. Proceedings of the 3rd International Conference on Inventive Material Science Applications.

[51] Bassoli E., Vincenzi L., Bovo M., Mazzotti C. Dynamic identification of an ancient masonry bell tower using a MEMS-based acquisition system. Proceedings of the IEEE Workshop on Environmental, Energy, and Structural Monitoring Systems Proceedings.

[52] Castañeda-Saldarriaga D.L., Alvarez-Montoya J., Martínez-Tejada V., Sierra-Pérez J. (2021). Toward Structural Health Monitoring of Civil Structures Based on Self-Sensing Concrete Nanocomposites: A Validation in a Reinforced-Concrete Beam. Int. J. Concr. Struct. Mater..

[53] Payawal J.M.G., Uy F.A.A., Carreon J.P.D. Data calibration of the actual versus the theoretical micro electro mechanical systems (MEMS) based accelerometer reading through remote monitoring of Padre Jacinto Zamora Flyover. Proceedings of the IEEE Conference on Technologies for Sustainability.

[54] Zonzini F., Zauli M., Mangia M., Testoni N., De Marchi L. HW-Oriented Compressed Sensing for Operational Modal Analysis: The Impact of Noise in MEMS Accelerometer Networks. Proceedings of the IEEE Sensors Applications Symposium.

[55] Lin C.-H., Kang C.-W., Yang C.-C., Wu C.-M., Huang C.-M. A cost effective three-axis accelerometer for building structure health monitoring. Proceedings of the IEEE Sensors Applications Symposium.

[56] Rosal J.E.C., Caya M.V.C. Development of Triaxial MEMS Digital Accelerometer on Structural Health Monitoring System for Midrise Structures. Proceedings of the IEEE 10th International Conference on Humanoid, Nanotechnology, Information Technology, Communication and Control, Environment and Management.

[57] Garcia A.M., Perez Aguilar A.N. Affordable Instrument Design for Seismic Monitoring, Early Warning Systems and Control Actions to Risk Mitigation. Proceedings of the 13th APCA International Conference on Automatic Control and Soft Computing.

[58] Sliti M., Boudriga N. Building Structural Health Monitoring: An FBG-based estimation of external vibrations. Proceedings of the 18th International Multi-Conference on Systems, Signals & Devices.

[59] Mahmud M.A., Bates K., Wood T., Abdelgawad A., Yelamarthi K. A complete Internet of Things (IoT) platform for Structural Health Monitoring (SHM). Proceedings of the IEEE 4th World Forum Internet Things.

[60] Chanv B., Bakhru S., Mehta V. Structural health monitoring system using IOT and wireless technologies. Proceedings of the International Conference on Intelligent Communication and Computational Techniques.

[61] Khoroshavin E.A. (2021). Dynamic tests and monitoring of the dynamic state of buildings and structures based on microseismic vibrations. Mag. Civ. Eng..

[62] Pentaris F.P., Stonham J., Makris J.P. A review of the state-of-the-art of wireless SHM systems and an experimental set-up towards an improved design. Proceedings of the Eurocon 2013.

[63] Stephan C. (2012). Sensor placement for modal identification. Mech. Syst. Signal Process..

[64] Zini G., Betti M., Bartoli G. (2022). A pilot project for the long-term structural health monitoring of historic city gates. J. Civ. Struct. Health Monit..

[65] Jeong S.-H., Jang W.-S., Nam J.-W., An H., Kim D.-J. (2020). Development of a Structural Monitoring System for Cable Bridges by Using Seismic Accelerometers. Appl. Sci..

[66] Kaya Y., Safak E. (2015). Real-time analysis and interpretation of continuous data from structural health monitoring (SHM) systems. Bull. Earthq. Eng..

[67] Giannoccaro N.I., Spedicato L., Foti D. A digital analysis of the experimental accelerometers data used for buildings dynamical identification. Proceedings of the IEEE Workshop on Environmental, Energy, and Structural Monitoring Systems.

[68] Pallarés F.J., Betti M., Bartoli G., Pallarés L. (2021). Structural health monitoring (SHM) and Nondestructive testing (NDT) of slender masonry structures: A practical review. Constr. Build. Mater..

[69] Amezquita-Sanchez J.P., Adeli H. (2016). Signal Processing Techniques for Vibration-Based Health Monitoring of Smart Structures. Arch. Comput. Methods Eng..

[70] Gaviria C., Montejo L. (2021). Unscented Kalman filter approach for tracking physical and dynamic properties of structures: Validation for multi-story buildings under seismic excitation. Struct. Monit. Maint..

[71] Emanov A.F., Maksimenko V.N., Sklyarov L.A. Technology of Diagnostics and Monitoring of State of Building Structures Based on the Microseismic Vibration Analysis. Proceedings of the International Forum on Strategic Technology.

[72] Pan H. (2022). Earthquake ground motion estimation for buildings using absolute floor acceleration response data. Earthq. Eng. Struct. Dyn..

[73] He Y., Chen H., Liu D., Zhang L. (2021). A Framework of Structural Damage Detection for Civil Structures Using Fast Fourier Transform and Deep Convolutional Neural Networks. Appl. Sci..

[74] Sanchez W.D., de Brito J.V., Avila S.M. (2020). Structural Health Monitoring Using Synchrosqueezed Wavelet Transform on IASC-ASCE Benchmark Phase I. Int. J. Struct. Stab. Dyn..

[75] Fujino Y., Siringoringo D.M., Ikeda Y., Nagayama T., Mizutani T. (2019). Research and Implementations of Structural Monitoring for Bridges and Buildings in Japan. Engineering.

[76] Flah M., Nunez I., Ben Chaabene W., Nehdi M.L. (2021). Machine Learning Algorithms in Civil Structural Health Monitoring: A Systematic Review. Arch. Comput. Methods Eng..

[77] Avci O., Abdeljaber O., Kiranyaz S., Hussein M., Gabbouj M., Inman D.J. (2021). A review of vibration-based damage detection in civil structures: From traditional methods to Machine Learning and Deep Learning applications. Mech. Syst. Signal Process..

[78] Ierimonti L., Cavalagli N., Venanzi I., García-Macías E., Ubertini F. (2021). A transfer Bayesian learning methodology for structural health monitoring of monumental structures. Eng. Struct..

[79] Huang S., Chao S., Huang J., Chang Y., Loh C. (2021). Estimation of story drift directly from acceleration records for post-earthquake safety evaluations of buildings. Earthq. Eng. Struct. Dyn..

[80] Avci O., Abdeljaber O., Kiranyaz S., Hussein M., Inman D.J. (2018). Wireless and real-time structural damage detection: A novel decentralized method for wireless sensor networks. J. Sound Vib..

[81] Zini G., Betti M., Bartoli G. (2022). A quality-based automated procedure for operational modal analysis. Mech. Syst. Signal Process..

[82] Carratu M., Espirito-Santo A., Monte G., Paciello V. (2020). Earthquake Early Detection as an IEEE1451 Transducer Network Trigger for Urban Infrastructure Monitoring and Protection. IEEE Instrum. Meas. Mag..

[83] Schultz A.E., Haro A.G., Liu M.K., Monzón A., Monzón H., Lanning F., Tola A. Influence of ground motion on performance of rc infill frames in the 2016 Ecuador earthquake. Proceedings of the 11th National Conference on Earthquake Engineering.

[84] MIDUVI, Norma Ecuatoriana de la Construcción—NEC Ministerio de Desarrollo Urbano y Vivienda. Quito, Ecuador, 2015. https://www.habitatyvivienda.gob.ec/documentos-normativos-nec-norma-ecuatoriana-de-la-construccion/.

